# The transcription factor ATF3 is upregulated during chondrocyte differentiation and represses cyclin D1 and A gene transcription

**DOI:** 10.1186/1471-2199-7-30

**Published:** 2006-09-19

**Authors:** Claudine G James, Anita Woods, T Michael Underhill, Frank Beier

**Affiliations:** 1 CIHR Group in Skeletal Development and Remodeling, University of Western Ontario, London, ON, Canada; 2 Department of Physiology and Pharmacology, Schulich School of Medicine and Dentistry, University of Western Ontario, London, ON, Canada; 3 Department of Cellular and Physiological Sciences, University of British Columbia, Vancouver, BC, Canada

## Abstract

**Background:**

Coordinated chondrocyte proliferation and differentiation are required for normal endochondral bone growth. Transcription factors binding to the cyclicAMP response element (CRE) are known to regulate these processes. One member of this family, Activating Tanscription Factor 3 (ATF3), is expressed during skeletogenesis and acts as a transcriptional repressor, but the function of this protein in chondrogenesis is unknown.

**Results:**

Here we demonstrate that *Atf3 *mRNA levels increase during mouse chondrocyte differentiation *in vitro *and *in vivo*. In addition, *Atf3 *mRNA levels are increased in response to cytochalasin D treatment, an inducer of chondrocyte maturation. This is accompanied by increased *Atf3 *promoter activity in cytochalasin D-treated chondrocytes. We had shown earlier that transcription of the cell cycle genes cyclin D1 and cyclin A in chondrocytes is dependent on CREs. Here we demonstrate that overexpression of ATF3 in primary mouse chondrocytes results in reduced transcription of both genes, as well as decreased activity of a CRE reporter plasmid. Repression of cyclin A transcription by ATF3 required the CRE in the cyclin A promoter. In parallel, ATF3 overexpression reduces the activity of a SOX9-dependent promoter and increases the activity of a RUNX2-dependent promoter.

**Conclusion:**

Our data suggest that transcriptional induction of the *Atf3 *gene in maturing chondrocytes results in down-regulation of cyclin D1 and cyclin A expression as well as activation of RUNX2-dependent transcription. Therefore, ATF3 induction appears to facilitate cell cycle exit and terminal differentiation of chondrocytes.

## Background

Growth and development of endochondral bones is controlled through the highly coordinated proliferation and differentiation of growth plate chondrocytes [1-3]. These processes are regulated by a large number of endocrine, paracrine and autocrine hormones and growth factors that, to a large part, act on chondrocyte cell surface receptors. The intracellular signaling pathways mediating these effects are not completely understood; however, over the last 10 years many of the key transcriptional regulators of chondrocyte differentiation have been identified. The *Sox9 *gene is required for the differentiation of mesenchymal precursor cells to chondrocytes and, together with the related L-Sox5 and Sox6 proteins, controls chondrocyte-specific gene expression [4,5]. Sox9 also inhibits terminal differentiation of chondrocytes to the hypertrophic phenotype [6]. In contrast, the *Runx2 *gene (also known as Cbfa1) is essential for differentiation of osteoblasts, but also promotes hypertrophic chondrocyte differentiation [5,7].

In addition to these key regulators of chondrocyte maturation, numerous other transcription factors have been implicated in this process. One example is the activating transcription factor/cyclicAMP response element-binding protein (ATF/CREB) family that is defined by the ability of its members to bind to the cyclicAMP response element (CRE) in target promoters. Mice with inactivation of the *Atf2 *gene display chondrodysplasia and reduced chondrocyte proliferation [8], a similar phenotype to transgenic mice overexpressing a dominant-negative form of CREB in cartilage [9]. We and others have shown that ATF2 and CREB regulate the transcription of the cell cycle genes cyclin D1 and cyclin A in chondrocytes through CRE-dependent mechanisms [10-15]. However, the ATF/CREB family contains additional members [16,17], some of which (such as ATF3) act as transcriptional repressors. These repressors could down-regulate CRE-dependent transcription and thus cause delay of cell cycle progression and/or promote cell cycle exit during terminal differentiation.

*Atf3 *expression has been shown to be induced by a large variety of cellular stresses, including radiation, DNA-damaging agents, adenoviral infection and others [18-22]. Recent evidence also suggests induction of ATF3 by a number of physiological stimuli such as growth hormone [23], transforming growth factor β[24] and ligands for several G-protein-coupled receptors [25]. In some instances (e.g. in certain heterodimers with other proteins or certain isoforms of ATF3), ATF3 has been shown to activate transcription [26,27]. However, in most cases ATF3 has been found to repress transcription of target genes [17]. Similarly, the effects of ATF3 on cellular physiology appear to be very context- and cell type-dependent; for example, ATF3 has been shown to both promote [27-29] and inhibit [21,30,31] cellular proliferation and cell cycle progression, and to have both pro- and anti-apoptotic effects [31,32].

We recently performed microarray analyses of an *in vitro *system of mouse chondrocyte differentiation in three-dimensional micromass cultures [33]. We now extended these studies and demonstrated that numerous ATF/CREB family members are expressed in chondrocytes. Most notably, ATF3 expression is markedly upregulated during chondrocyte differentiation. Validation of ATF3 expression and functional studies strongly suggest an important role for ATF3 in cell cycle exit and terminal differentiation.

## Results

### Atf3 mRNA levels increase during chondrocyte differentiation

**Figure 1:**
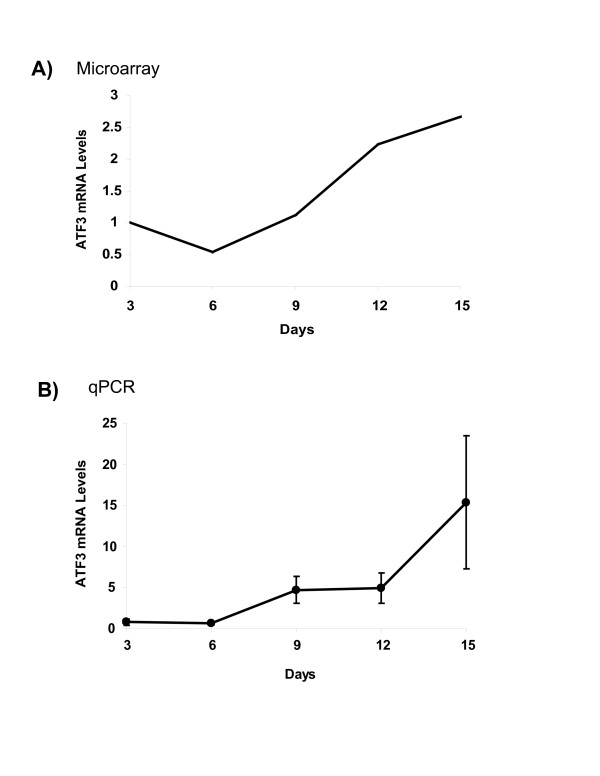
***Atf3 *expression is upregulated during chondrocyte differentiation *in vitro***. *Atf3 *mRNA expression during chondrogenic differentiation of mouse embryonic limb bud cells in micromass culture was examined by microarray (A) and real-time PCR (B) analyses (different cell isolations were used for the experiments in A and B). Microarray data sets represent averages from three independent experiments. Real-time data were normalized to *Gapdh *levels and present average and SEM from four independent experiments. Both approaches show similar expression patterns with a strong increase in *Atf3 *expression during micromass differentiation.

Based on the prominent role of ATF2 (gene name *Atf2*) and CREB (*Creb1*) in cartilage development, we analyzed the expression of ATF-CREB family members in our transcriptional profiles of differentiating micromass cultures [33]. Numerous family members were expressed during micromass differentiation (Table 1). Most genes, including *Atf2 *and *Creb1*, did not display marked changes in expression during micromass differentiation, and no genes were down-regulated two-fold or more. However, *Atf3*, *Atf5*, *Atf6 *and *Creb3l1 *genes displayed upregulation towards the end of the time course. The strongest effects were observed for *Atf3 *transcript levels that increased 4.7-fold from day 3 to 15 of micromass culture (Fig. 1A). *Atf5*, *Atf6 *and *Creb3l1 *mRNA levels increased 4.3-, 2.5- and 3.5-fold over the same period, respectively. Because it showed the largest increase, we focused subsequent studies on the *Atf3 *gene. We performed real-time PCR analyses of independent micromass cultures from mouse embryonic limb bud cells and demonstrated a strong increase of *Atf3 *mRNA expression during micromass differentiation, thus validating the microarray data (Fig. 1B).

**Figure 2:**
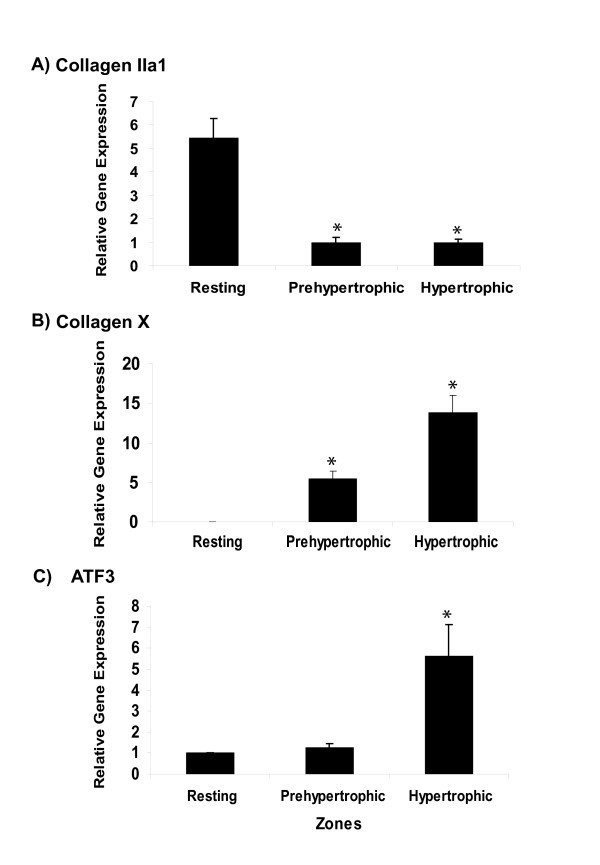
***Atf3 *expression is upregulated during chondrocyte differentiation *in vivo***. Tibia isolated from embryonic day 15.5. mice were microdissected into the resting/proliferating, prehypertrophic and hypertrophic areas, and RNA was isolated directly out of cartilage. Expression of type II collagen II (*Col2a1*), type X collagen (*Col10a1*) and *Atf3 *was examined by real-time PCR analyses and normalized to *Gapdh *expression. All data represent averages and SEM from four independent experiments (*: p < 0.05). *Col2a1 *was strongly expressed in the resting/proliferating area and declined subsequently (A). *Col10a1 *was virtually undetectable in the resting/proliferating area and strongly induced in the prehypertrophic and hypertrophic areas (B). *Atf3 *was already expressed in the resting/proliferating area, but expression was significantly increased in the more mature zones of the growth plate.

To examine the expression of *Atf3 *during chondrocyte differentiation *in vivo*, we dissected tibiae from embryonic day 15.5 CD1 mice into the resting/proliferating, prehypertrophic and hypertrophic areas (at this stage, CD1 tibiae are not vascularized yet; the center of the bone is thus still comprised of hypertrophic cartilage). RNA was directly isolated from dissected tibiae, without subculturing of cells. Real-time PCR analyses demonstrated expression of collagen II (*Col2a1*) in the resting/proliferating area that declines significantly in the prehypertrophic and hypertrophic areas (Fig. 2A). In contrast, collagen X (*Col10a1*) mRNA was virtually undetectable in the resting/proliferating area and increased strongly in the other zones (Fig. 2B). These expression profiles of known markers of early and late chondrocyte differentiation confirmed efficient separation of the different growth pate zones by microdissection. *Atf3 *mRNA levels increased more than five-fold from the resting/proliferating to the hypertrophic zone (Fig. 2C). *Atf3 *expression thus displayed a similar pattern as *Col10a1*, although the induction was less pronounced for *Atf3*.

### Atf3 is upregulated by cytochalasin D, an inhibitor of actin polymerization

**Figure 3:**
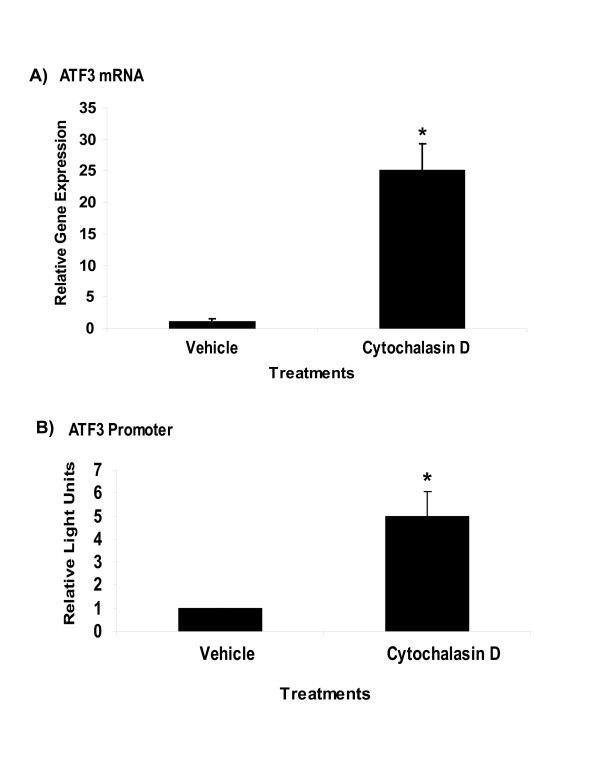
**Inhibition of actin polymerization induces *Atf3 *expression through transcriptional mechanisms**. A) Primary chondrocytes were cultured for 24 hours with DMSO (control) or 1 μM cytochalasin D before harvest and RNA isolation. Real-time PCR demonstrated marked induction of *Atf3 *mRNA levels by cytochalasin D. Data represent averages and SEM from three independent experiments, normalized to *Gapdh *(*: p < 0.05). B) Primary chondrocytes were transfected with an *Atf3 *promoter plasmid and pRLCMV, followed by incubation for 24 hours with DMSO (control) or 1 μM cytochalasin D. Cells were then harvested, firefly luciferase activity was measured and normalized to Renilla luciferase activity. Data represent averages and SEM from three independent experiments, performed in quadruplicate each (*: p < 0.05). Cytochalasin D induced *Atf3 *promoter activity.

We had shown earlier that inhibition of actin polymerization by cytochalasin D promotes chondrogenic differentiation [34,35]. Thus, we examined whether cytochalasin D affects *Atf3 *expression. Indeed, incubation of primary chondrocytes with cytochalasin D caused a more than 30-fold induction of *Atf3 *mRNA levels (Fig. 3A). To examine whether this upregulation is due to transcriptional effects, primary chondrocytes were transfected with an *Atf3 *promoter – firefly luciferase construct. Cytochalasin D treatment resulted in five-fold upregulation of *Atf3 *promoter activity (Fig. 3B). Thus, cytochalasin D induces *Atf3 *through transcriptional mechanisms, at least in part.

### ATF3 represses SOX9-dependent and increases RUNX2-dependent transcription

**Figure 4:**
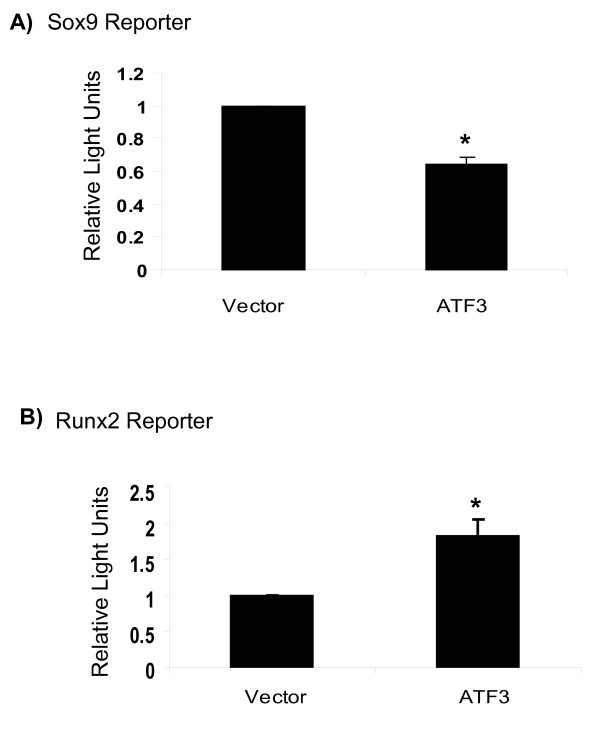
**ATF3 suppresses Sox9-dependent transcription and stimulates Runx2-dependent transcription**. Primary chondrocytes were cotransfected with SOX9 (A) or RUNX2 (B) reporter plasmids, pcDNA3 (control) or an ATF3 expression vector, and pRLCMV. 24 hours after transfection, cells were harvested, firefly luciferase activity was measured and normalized to Renilla luciferase activity. Data represent averages and SEM from three independent experiments, performed in quadruplicate each (*: p < 0.05). ATF3 overexpression suppressed SOX9-dependent transcription and stimulated RUNX2-dependent transcription.

We next asked whether increased expression of *Atf3 *affects chondrocyte-specific gene expression. Primary chondrocytes were cotransfected with reporter vectors for SOX9 and RUNX2 activity and an ATF3 expression vector. Overexpression of ATF3 resulted in an approximately 40 % reduction in SOX9-dependent transcription (Fig. 4A) and an 80 % increase in RUNX2-dependent transcription (Fig. 4B), suggesting that upregulation of ATF3 can promote the transition from proliferating to hypertrophic chondrocytes.

### ATF3 represses transcription of the cyclin D1 and cyclinA genes

**Figure 5:**
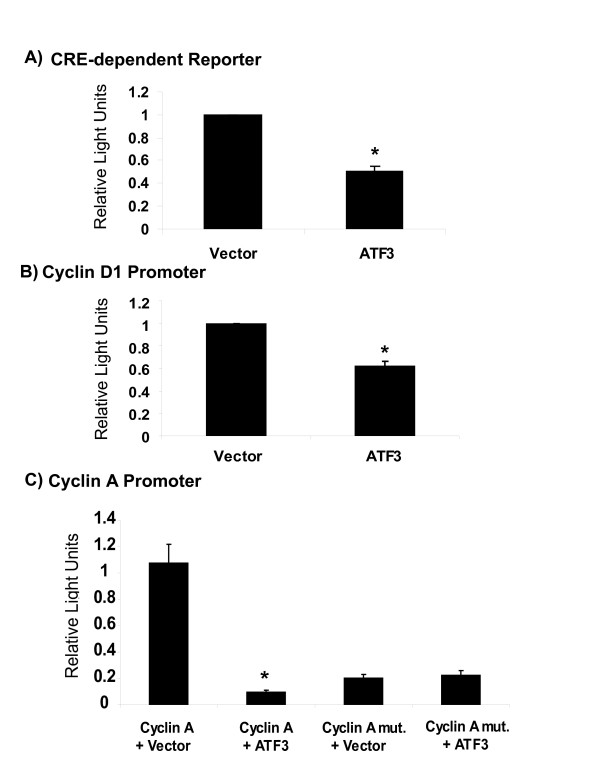
**ATF3 suppresses CRE-dependent transcription**. Primary chondrocytes were cotransfected with a CRE reporter (A), a cyclin D1 promoter (-1745CD1LUC, B) or cyclin A promoter plasmids (p707cycAluc and p707cycAlucMut; C), pcDNA3 (control) or an ATF3 expression vector, and pRLCMV. 24 hours after transfection, cells were harvested, firefly luciferase activity was measured and normalized to Renilla luciferase activity. Data represent averages and SEM from three independent experiments, performed in quadruplicate each (*: p < 0.05). ATF3 overexpression suppressed the CRE reporter, cyclin D1 and cyclin A wild type promoters significantly, whereas the mutant cyclin A construct showed no response to ATF3 overexpression.

ATF3 acts as a transcriptional repressor through the CRE. We had demonstrated a requirement for the CRE for maximal activity of the cyclin D1 and cyclin A promoters in chondrocytes [11,12,14,15]. We thus examined the effects of ATF3 overexpression on CRE-dependent transcription and the activity of these two cyclin promoters. Transient transfection of an ATF3 expression plasmid decreased the activity of the CRE-dependent reporter by approximately 50 % (Fig. 5A) and cyclin D1 promoter activity by 38 % (Fig. 5B). ATF3 overexpression most strongly repressed cyclin A promoter activity which decreased to less than 10% of control values (Fig. 5C). To determine whether the effects of ATF3 on cyclin A transcription were mediated by the CRE, we performed parallel experiments using a cyclin A promoter construct with a mutated CRE (Fig. 5C). ATF3 overexpression did not affect activity of this promoter fragment, suggesting that ATF3 represses cyclin A transcription directly through the CRE.

## Discussion

This study is the first to demonstrate an important role of ATF3 in the control of endochondral bone growth and skeletal development. Our data demonstrate upregulation of *Atf3 *during chondrogenic differentiation *in vivo *and *in vitro*. *Atf3 *expression is known to be induced by a number of different cellular stressors, including hypoxia [22]. Hypoxia has been demonstrated in the cartilage growth plate *in vivo *[36], raising the possibility that low oxygen tension is one of the physiological inducers of ATF3 in developing cartilage. Interestingly, two of the other ATF/CREB family members found to be upregulated in our studies, ATF6 and CREB3L1 (also known as OASIS), have also been shown to be induced by cellular stress (in particular endoplasmatic reticulum stress) [37-41]. These data suggest that upregulation of stress-responsive transcription factors is a common theme during late stage chondrogenic differentiation in micromass culture. Since *Atf3 *transcript levels display similar upregulation during chondrocyte differentiation *in vivo*, this pattern does not appear to be an artifact of the micromass culture system, but to reflect the physiological processes of cartilage development.

ATF2 and CREB, the prototype members of the ATF-CREB family, have been shown to play crucial roles in chondrocyte cell cycle gene expression and proliferation [42,43]. While their activities are essential to ensure adequate rates of proliferation, physiological bone development also requires regulated cell cycle exit and onset of postmitotic chondrocyte differentiation. These requirements necessitate tight control of ATF2 and CREB activity that can be achieved through several mechanisms. For example, both transcription factors are regulated by posttranslational modifications such as phosphorylation. Changes in phosphorylation status could therefore repress their activity when chondrocytes exit the proliferative phase. For example, we and others have shown that extracellular signals such as Transforming Growth Factor β, Parathyroid Hormone-related Peptide and prostaglandins induce phosphorylation of ATF2 and CREB in chondrocytes, respectively [10,13,44]. Our data presented here also suggest that *Atf2 *and *Creb1 *mRNA levels do not change during chondrocyte differentiation, suggesting that they are regulated at the posttranscriptional level (e.g. through phosphorylation as discussed above and/or by regulation of protein stability). Another possibility to limit ATF2/CREB activity is the expression of transcriptional repressors that compete with them for binding elements on target genes. One such repressor is ATF3 [26].

Our data show that *Atf3 *mRNA expression is markedly upregulated during chondrocyte differentiation in a three-dimensional micromass culture system *in vitro *and in mouse embryonic tibiae *in vivo*. In addition, *Atf3 *is strongly induced in response to cytochalasin D, an actin-modifying drug that induces early [34,35,45,46] and late (Woods and Beier, in prep.) chondrocyte differentiation. Our transient transfection assays demonstrate that the induction of *Atf3 *expression by cytochalasin D occurs, at least in part, at the transcriptional level; however, induction of mRNA levels is markedly higher than stimulation of promoter activity. This could be due to posttranscriptional mechanisms such as increased mRNA stability as has been described for *Atf3 *before [47]. Alternatively, transcriptional activation of the endogenous *Atf3 *gene through promoter/enhancer elements not present in our reporter plasmid is possible.

The expression pattern of *Atf3 *is therefore consistent with a function during cell cycle exit and terminal differentiation. Furthermore, our data suggest that ATF3 represses CRE-dependent transcription, for example the activity of the cyclin D1 and cyclin A promoters. We have shown earlier that the activities of these promoters correlates with the corresponding protein levels in chondrocytes [11-13]. Induction of ATF3 should therefore result in reduced levels of these cyclins and other ATF2/CREB targets. The cyclin A promoter showed a much stronger response to ATF3 overexpression than the cyclin D1 promoter and the CRE reporter. The most plausible explanation for this difference is that the cyclin A promoter, in addition to direct regulation through the CRE, is also controlled by upstream cell cycle proteins such as cyclin D1 and E2F [48,49]. Reduced cyclin D1 levels and E2F activity in response to ATF3 overexpression could therefore contribute to the observed strong downregulation of cyclin A transcription by ATF3.

Mutation of the CRE in the cyclin A promoter completely abolished the response to ATF3 overexpression, providing strong evidence that the effects of ATF3 on cyclin A transcription are mediated by direct binding to the CRE. Interestingly, overexpression of ATF3 repressed cyclin A promoter more than mutation of CRE. This suggests that binding of ATF3 to the CRE also suppresses the activity of other elements in the promoter, for example the E2F response element discussed above.

**Figure 6:**
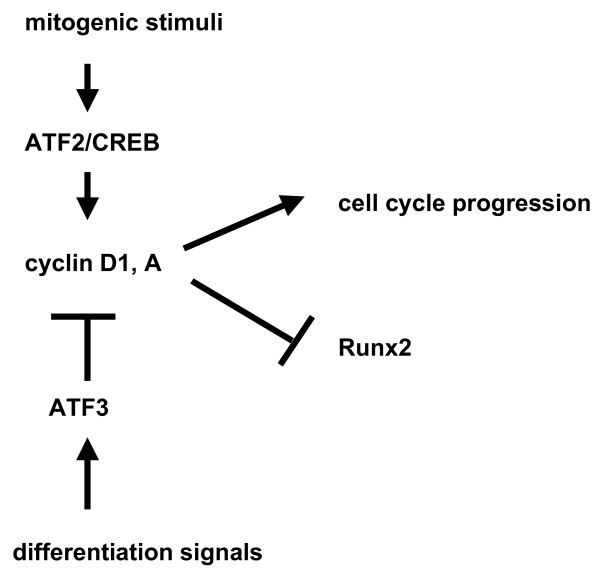
**Model for ATF3 action in chondrocyte differentiation**. We suggest that induction of ATF3 by differentiation stimuli antagonizes the CRE-dependent expression of cyclin D1 and cyclin A, which are induced by mitogenic stimuli through ATF2 and CREB. Reduced cyclin-dependent kinase activity and hypophosphorylation of pocket proteins in response to ATF3 upregulation then results both in cell cycle exit and in increased activity of Runx2, promoting chondrocyte differentiation.

In growth plate physiology, Sox9 suppresses and Runx2 promotes chondrocyte hypertrophy. In this study, we observed repression of Sox9 activity and enhancement of Runx2 activity in response to ATF3 overexpression, in agreement with the postmitotic role of ATF3 suggested by us. However, the mechanism(s) involved remain to be elucidated. One possibility is that ATF3, indirectly or directly, regulates expression of Sox9 and/or Runx2, or required cofactors. A second possibility is that ATF3 physically interacts with each or both of these factors, thereby modulating their activity. Both Sox9 and Runx2 are known to undergo protein-protein interactions with many other transcription factors. For example, Sox9 has been shown to interact with c-Maf [50], steroidogenic factor 1 [51] and β-catenin [52]. Runx2 has been shown to interact with, among others, Nrf2 [53] and Smad [54] proteins. Most notably, Runx2 has been demonstrated to bind to ATF4, another member of the ATF/CREB family, although this interaction appears to be indirect [55]. A third possibility is that the effects of ATF3 on Sox9 and Runx2 activity are secondary to ATF3's effect on cell cycle progression. It has been shown that Runx2 activity fluctuates throughout the cell cycle and is controlled by the cell cycle machinery [56-59]. For example, cyclin D1 together with cyclin-dependent kinase 4 has been shown to promote ubiquitination and degradation of Runx2 [60]. Repression of cyclin D1 expression by ATF3 would thus result in stabilization of Runx2 and increased differentiation. Our model for the effects of ATF3 on chondrocyte cell cycle gene expression and Runx2 activity is illustrated in Fig. 6. To our knowledge, no cell cycle control mechanisms for Sox9 activity have been reported, but are conceivable.

## Conclusion

In summary, this study identifies ATF3 as a novel player in the complex networks controlling growth plate chondrocyte proliferation and differentiation. Induction of *Atf3 *expression by differentiation stimuli is likely to contribute to the coordination of cell cycle exit and hypertrophic differentiation during endochondral bone growth. Analyses of the *in vivo *roles of ATF3 in endochondral ossification and identification of the physiological inducers and target genes of ATF3 during skeletogenesis will be important steps towards a better understanding of cartilage development.

## Methods

### Materials

Timed-pregnant CD1 mice (embryonic day 11.5 [E11.5] or E15.5) were obtained from Charles River. Real-time PCR reagents were obtained from Applied Biosystems. Cell culture reagents and the pcDNA3 plasmid were from Invitrogen, and cytochalasin D was purchased from Calbiochem. Fugene 6 was obtained from Roche, the dual luciferase assay and the plasmid pRLCMV were from Promega. The CRE reporter plasmid was from Stratagene. The SOX9 reporter plasmid has been described [61]; the RUNX2 reporter plasmid was constructed in a similar fashion (8 copies of a Runx2 binding site cloned upstream of a minimal osteocalcin promoter in pGl3basic [Promega]). The *Atf3 *promoter plasmid [23], the ATF3 expression plasmid [62], the cyclin D1 promoter plasmid -1745CD1Luc [63] and the cyclin A promoter plasmids pcycAluc707 and pcycAluc707mut [64] plasmid were generously provided by Drs. J. Schwartz (University of Michigan), D. Steiner (University of Chicago), R. Pestell (Georgetown University) and K. Oda (Science University of Tokyo), respectively.

### RNA isolation, microarray analyses and real-time PCR

Sample generation, RNA isolation, microarrays and bioinformatics analyses for these data have been described recently [33]. For microdissections, embryonic day 15.5 tibiae were dissected into the resting/proliferating, prehypertrophic and hypertrophic areas under a stereomicroscope, and RNA was isolated following the RNeasy ^® ^Lipid Tissue Extraction protocol from Qiagen (Mississauga). RNA integrity was verified using the Agilent 2100 Bioanalyzer. Real-time PCR was performed as described, using Assays-on-Demand (Applied Biosystems) and *Gapdh *for standardization [33,34,65]. All reactions were run in quadruplicate from at least three independent experiments.

### Micromass cultures and primary chondrocyte cultures

Micromass cultures from E11.5 mouse limb buds and isolation of primary murine chondrocytes from E15.5 long bones were performed as recently described [33,66]. Micromass cultures were differentiated for 15 days, and samples were harvested every three days for RNA isolation. Primary chondrocytes were plated in monolayer culture in medium containing 10 % fetal bovine serum and incubated for 24 hours with DMSO (control) or 1 μM cytochalasin D before harvest for RNA as described [35].

### Transient transfections and luciferase assays

Primary chondrocytes in monolayer culture were transiently transfected using Fugene 6 as described [14,35,67]. For *Atf3 *promoter analyses, cells were transfected with the *Atf3 *promoter plasmid and pRLCMV for standardization. After transfection, cells were incubated with DMSO or 1 μM cytochalasin D for 24 hours before harvesting for luciferase assays. For cotransfections, cells were transfected with the respective promoter plasmid (cyclin D1, cyclin A or the CRE, SOX9 or RUNX2 reporters), pRLSV40 and empty expression vector (pcDNA3) or the ATF3 expression vector. Cells were harvested 24 hours after transfection. Firefly luciferase activity was determined and normalized to Renilla luciferase activity. All data shown present averages and SEM from three independent experiments done in quadruplicate each.

### Statistical analyses

Statistical significance of real-time PCR and luciferase results was determined by two-way ANOVA with Bonferroni post-test using GraphPad Prism version 3.00 for Windows, GraphPad Software, San Diego California USA.

## Abbreviations

ATF, activating transcription factor; CRE, cyclic AMP response element, CREB, CRE-binding protein; PCR, polymerase chain reaction

## Authors' contributions

CGJ and AW performed experiments and contributed to the writing of the manuscript. TMU provided input into the design of the study and the manuscript. FB conceived of the study, performed selected experiments and contributed to the writing of the manuscript. All authors read and approved the final manuscript.

## Figures and Tables

**Table 1:** Microarray Expression Profiles of ATF/CREB Family Members

		Normalized Signal Intensity Values				
Genbank	Gene Symbol	Day 3	Day 6	Day 9	Day 12	Day 15

NM_007497	Atf1	0.62	0.87	1.13	1.27	1.158
BM119623	Atf2	1.10	0.95	0.98	1.00	0.91
BC019946	Atf3	0.84	0.42	0.96	3.40	3.92
AV314773	Atf4	0.99	0.87	1.03	1.42	1.03
AF375476	Atf5	0.40	0.74	1.16	2.54	1.735
AV270913	Atf6	0.74	0.73	1.01	1.75	1.818
BC026483	Atf7	1.09	1.07	0.95	1.08	0.875
NM_016767	Batf	0.90	0.97	1.01	1.04	1.058
NM_009952	Creb1	1.17	0.88	0.76	0.77	0.77
BG070002	Creb3	0.97	1.07	1.00	0.99	0.94
BC013534	Crebl1	1.58	1.21	1.01	0.91	0.860
BC016447	Creb3l1	0.30	0.71	1.09	1.34	1.037
BC010786	Creb3l3	0.94	1.03	1.15	0.99	1.002
BC022605	Creb3l4	0.92	0.93	1.08	0.86	0.862
AI467599	Crem	1.21	1.09	0.90	0.79	0.890
